# Defining the biological functions and clinical significance of AKR1C3 in gastric carcinogenesis through multiomics functional analysis and immune infiltration analysis

**DOI:** 10.7150/jca.94228

**Published:** 2024-03-17

**Authors:** Yongfu Shao, Xuan Yu, Keshu Shan, Jianing Yan, Guoliang Ye

**Affiliations:** 1 Health Science Center, Ningbo University, Ningbo, Zhejiang, 315211, China.; 2 Department of Gastroenterology, the First Affiliated Hospital of Ningbo University, Ningbo, Zhejiang, 315020, China.

**Keywords:** AKR1C3, gastric cancer, biomarker, multiomics, immune infiltration

## Abstract

**Background:** Human aldo-keto reductase family 1 member C3 (AKR1C3) is an important molecule that participates in multiple physiological metabolic processes. However, its expression, biological functions and clinical significance in gastric carcinogenesis are unclear.

**Methods:** We collected data from several public data portals and clinical samples and systematically analyzed the clinical significance of tissue and plasma AKR1C3 expression. Then, we filtered prognostic risk factors and established novel prognosis-related nomogram models for predicting overall survival time and postoperative recurrence risk. The application value of the nomogram models was further assessed using clinical samples. Moreover, we explored the potential biological functions of AKR1C3 in gastric carcinogenesis and metastasis through multiomics functional analysis and immune infiltration analysis.

**Results:** AKR1C3 levels were reduced in cancer tissue but increased significantly in the plasma of GC patients; AKR1C3 expression in either sample type was closely associated with multiple clinicopathological characteristics. By combining clinicopathological factors and AKR1C3 levels, two novel nomogram models were developed to predict overall survival time and postoperative recurrence risk. Multiomics functional analysis revealed that when its expression is dysregulated, AKR1C3 can widely participate in gene expression regulation through multiple regulatory modes at the gene, RNA and protein levels and exert various crucial biological effects in carcinogenesis and metastasis. Moreover, AKR1C3 expression was correlated with the infiltration of several immune cell types, and AKR1C3 was predicted to interact with several clinical drugs.

**Conclusion**: Dysregulated AKR1C3 expression is related to gastric carcinogenesis and immunotherapy response and is a promising biomarker and effective biotherapy target in GC.

## Introduction

Gastric cancer (GC) is the fifth leading cause of cancer-related morbidity and the fourth leading cause of cancer-related mortality, imposing a heavy economic burden on the healthcare system and society worldwide 1, 2. Nevertheless, as the symptoms of early GC are often nonspecific, the vast majority of GC patients are diagnosed at an advanced stage with several complications and a poor prognosis 3, 4. Clinicians still lack satisfactory specific and sensitive biomarkers for GC screening and diagnosis, especially in the early stage 5. Moreover, despite the development of minimally invasive surgery techniques and chemotherapy, radiation therapy and combination treatments, the therapeutic effect for advanced GC is still limited 6, 7. Breakthroughs in immunotherapy have dramatically transformed the treatment landscape; however, the complex tumor microenvironment and immune tolerance often lead to anticancer drug resistance and tumor recurrence 8, 9. There are still clinical challenges regarding the screening of early gastric cancer and biotherapy treatment of advanced gastric cancer.

Human aldo-keto reductase family 1 member C3 (AKR1C3) is a key enzyme in reducing 5α-dihydrotestosterone (5α-DHT) to 5α-androstane-3α,17β-diol (3α-diol) and oxidizing 3α-diol to androsterone, playing critical roles in regulating the metabolic processes of diverse steroid hormones and biological processes 10, 11. AKR1C3 is an important molecule that participates in multiple physiological functions and is associated with the progression of several human diseases 12. However, its expression, biological functions and clinical significance in gastric carcinogenesis are not yet known.

In this study, we first collected data from several public data portals and clinical samples and systematically analyzed the clinical significance of tissue and plasma AKR1C3 expression in the progression of gastric cancer. Then, we filtered prognostic risk factors and established novel prognosis-related nomogram models for overall survival time and postoperative recurrence risk prediction. The application value of nomogram models was further assessed using clinical tissue samples and plasma samples. Moreover, we explored the potential biological functions of AKR1C3 in gastric carcinogenesis and metastasis through multiomics functional analysis and immune infiltration analysis. In addition, the downstream regulatory mechanism- competing endogenous RNA (ceRNA) mechanism was also investigated to establish molecule regulatory network. Our data reveal that dysregulation of AKR1C3 is related to gastric carcinogenesis and the response to immunotherapy and is a promising biomarker and effective biotherapy target in GC.

## Methods and Materials

### Patients and clinical specimens

UCSC Xena (https://xena.ucsc.edu/) was used to extract pancancer transcriptome data, and clinical information was obtained from The Cancer Genome Atlas (TCGA) database (https://genome-cancer.ucsc.edu/). Normalized RNA-seq data (version 7) from the Genotype-Tissue Expression (GTEx) data portal (https://www.gtexportal.org/home/index.html) were downloaded.

Tissue and peripheral plasma samples used in this study were obtained from the First Affiliated Hospital of Ningbo University, China, between 2015 and 2020. GC tissues and paired adjacent normal tissues (5 cm away from the edge of the tumor) were collected from 78 patients who underwent surgical procedures. Furthermore, 37 healthy gastric mucosa (HGM), 34 chronic gastritis (CG) and 28 gastric dysplasia (GD) samples were collected as biopsy specimens. All cell lines were authenticated by short tandem repeat profiling, and the last authentication was in April, 2023. Mycoplasma contamination testing was regularly conducted and no mycoplasma contamination was confirmed. A total of 83 GC patient plasma samples were obtained before and two weeks after surgery, and 83 healthy plasma samples were also obtained. Tissue samples were immediately dissected and soaked in RNA conservative solution (Bioteke, Beijing, China) after removal and preserved at -80°C for further use. Each patient signed informed written consent prior to gastroscopy or surgery. The ethics committee of Ningbo University (IRB No. 20120303) approved this study.

### Genomic alterations of AKR1C3 across cancers

The cBioPortal database (http://www.cbioportal.org/) was used to analyze gene alterations of AKR1C3 in the stomach adenocarcinoma (TCGA, PanCancer Atlas) dataset 13. The genetic alterations and mutation site information of AKR1C3 were extracted via the “Oncoprint”, “Cancer Type Summary”, and “Mutations” modules 14.

### Distinguishing expression and prognostic value identification

AKR1C3 expression levels from the TCGA cohort and normal tissues in the GTEx cohort were compared by Student's t test. The relationship between the expression level of AKR1C3 and mismatch repair (MMR) was detected in the TCGA cohort 15. Next, Kaplan‒Meier plotter (http://kmplot.com/analysis/) 16 was used to evaluate the correlation between AKR1C3 expression and survival time from several databases, including the Gene Expression Omnibus and TCGA, to determine the prognostic value of AKR1C3 expression in GC.

### Quantitative real-time polymerase chain reaction detection

Total RNA was extracted from samples by TRIzol (Ambion, Carlsbad, USA) and reverse transcribed to cDNA using the GoScript Reverse Transcription (RT) System (Promega, Madison, USA). Then, quantitative real-time polymerase chain reaction (qRT‒PCR) detection was carried out using GoTaq qPCR Master Mix (Promega) on an Mx3005P Real-Time PCR System (Stratagene, La Jolla, CA, USA). The qRT‒PCR cycling conditions were as follows: 95 ºC for 5 min, followed by 45 cycles of 94 °C for 15 s, 55 °C for 30 s, and 72 °C for 30 s. GAPDH mRNA was selected to normalize AKR1C3 expression. The primers used for qRT‒PCR were as follows: AKR1C3: forward, 5'-GCCTGTATTGGGATTTGGCACCTAT-3', reverse, 5'-GCGGAACCCAGCTTCTATTGCTAA-3. GAPDH: forward, 5′-ACCCACTCCTCCACCTTTGAC-3′, reverse, 5′-TGTTGCTGTAGCCAAATTCGTT-3′. The expression level of AKR1C3 was calculated via the Δ*C*t method (Δ*C*t=*C*t _AKR1C3_ - *C*t _GAPDH_). A higher Δ*C*t value indicates a lower AKR1C3 expression level.

### Validation of the prognostic and diagnostic values of AKR1C3

Kaplan‒Meier curves were built using GraphPad Prism 9.0 (GraphPad Software, USA) to validate the prognostic value of AKR1C3. Receiver operating characteristic (ROC) curves were built, and the area under the curve (AUC), sensitivity and specificity values were calculated to evaluate the diagnostic value.

### Establishment of prognostic and diagnostic nomogram models

Related clinicopathologic characteristics were integrated, and univariate and multivariable Cox models were used to construct an overall survival (OS) time nomogram model via the R package rms (version 6.2-0) in the TCGA cohort. The concordance index (C-index) was calculated to assess the discrimination of the nomogram, and the model was calibrated by contrasting calibration plots to predict the 1-, 3- and 5-year OS of GC patients. Decision curve analysis (DCA) 17 was performed to evaluate the clinical net benefit. Likewise, multivariate logistic regression was performed to identify independent predictors using the plasma samples. Nomograms were built using a logistic regression model with the R package rms (version 6.2-0). The C-index was calculated to assess the discrimination ability of the nomogram.

### Multiomic functional analysis of AKR1C3

GeneMANIA (https://genemania.org/) 18 was used to analyze the interaction network of AKR1C3 at the gene level. Then, the genes from the interaction network were input into the Search Tool for the Retrieval of Interacting Genes/Proteins (STRING) website (https://string-db.org/) to build a protein‒protein interaction (PPI) network. A combined score ≥ 0.7 was considered to indicate a significant PPI pair 19. KEGG pathway enrichment analysis and gene ontology (GO) classification based on the nodes in the PPI network were further performed via the R packages “clusterProfiler” and “ggplot2”. *P* value < 0.05, min enrichment > 3, and min overlap > 3 were considered significant.

The target miRNAs of AKR1C3 were obtained from TargetScan 8.0 (https://www.targetscan.org/vert_80/) 20. The “stomach” parameter in the tissue module and the “validated as experiments” parameter were selected to choose the target miRNAs. DIANA-miRPath v4.0 (https://diana-lab.e-ce.uth.gr/app/miRPathv4) was used for the target-based functional analysis of miRNAs 21. Tarbase v8.0, miRbase-v22.1, and KEGG databases were utilized, and Tarbase targets, gene union data, and classic analysis data were utilized to extract the mRNAs binding to these miRNAs associated with pathways in cancer (P<0.05). StarBase v3.0 (https://rnasysu.com/encori/) was used to predict lncRNAs and circRNAs binding to miRNAs, AKR1C3 mRNA-RBP interactions, RBP-lncRNA interactions, and RBP-circRNA targets 22. The screening criteria were mammal, human, hg19, strict stringency (≥3) of CLIP-Data, and medium stringency of Degradome-Data to filter the target lncRNAs and circRNAs.

The RBP-disease module was used to identify stomach-related AKR1C3 mRNA-RBPs. With the threshold of high stringency (≥3) for the CLIP-Data and ≥5 for the pancancer data, the target RBP-lncRNA pairs or circRNAs were filtered. The differentially expressed RBP-lncRNA pairs (*P*< 0.05 in GC TCGA database) were finally selected. Cytoscape v3.8.0 software was used to build networks.

### Immune infiltration analysis of AKR1C3

Relationships between prognosis-related genes and the abundance of several infiltrating immune cells in GC were explored using the R package GSVA (version 1.34.0). The stromal, immune, estimate scores were computed via the R package estimate (version 1.0.13) with the default parameters 23. The Tumor Immunization Single Cell Center (TISCH, http://tisch.comp-genomics.org/home/) is a single-cell RNA sequencing database of the tumor microenvironment used to reveal the purity and immune infiltration of GC 24. TISIDB (http://cis.hku.hk/TISIDB/index.php) is a web portal integrating multiple heterogeneous data types that was performed for detecting GC and immune system interactions 25. Pearson's correlation analysis was used to determine the correlation between AKR1C3 expression and indicators (*P*<0.05).

### Statistical analysis

Analyses in this study were performed via R software (version 3.2.3) or GraphPad (version 9.0) and their support packages as mentioned before. *P*<0.05 was considered to indicate significance.

## Results

### Dysregulated expression of AKR1C3 between various human tumor and normal tissues

**Figure 1:**
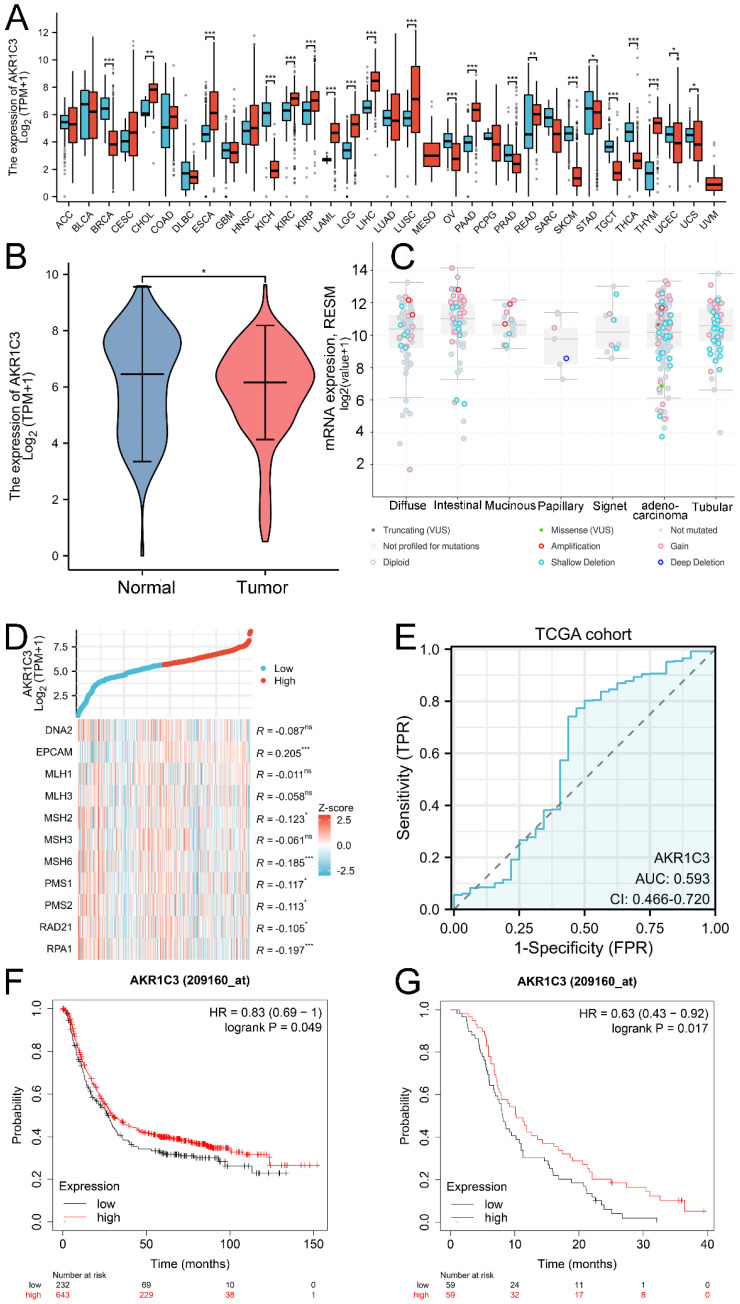
** Analysis of AKR1C3 expression and clinical significance in public databases.** A: Differential expression of AKR1C3 across cancers. B: AKR1C3 is downregulated in GC tissues in the TCGA and GTEx cohorts. C: Mutation types of AKR1C3 in the subtypes of GC. D: Coexpression heatmap. E: ROC curve of AKR1C3 for distinguishing GC samples from adjacent normal tissues in the TCGA cohort. F: Lower AKR1C3 expression was associated with poor OS in the TCGA cohort. G: Lower AKR1C3 expression was associated with poor OS in the GSE14210 cohort. (*P <0.05, **P <0.01, ***P < 0.001).

To reveal and compare the expression levels of AKR1C3 in normal human tissues and their corresponding tumor tissues, big data from public databases were analyzed. As shown in Figure 1A, differential expression of AKR1C3 was common across cancers. Compared with that in other human tissues, the expression of AKR1C3 in normal gastric mucosa is relatively high but decreases abnormally during carcinogenesis. Then, to further reveal the clinical significance of dysregulated AKR1C3 expression in gastric cancer, data for 414 GC tissue samples and 36 paired adjacent normal tissue samples were downloaded from the TCGA database, and data for 174 normal tissues were downloaded from the GTEx database. As shown in Figure 1B, compared with those in normal gastric tissues, AKR1C3 expression levels were significantly downregulated in GC tissues (*P* =0.036). As the clinical information of the TCGA cohort is shown in Supplementary Table 1, AKR1C3 expression levels in GC tissue were closely related to histologic grade. Moreover, mutation of the AKR1C3 gene in GC was detected via the cBioPortal platform. Amplification was the main mutation type for AKR1C3, and the mutation sites of AKR1C3 are displayed in Supplementary Figure 1. The mutation types of AKR1C3 in the subtypes of GC are shown in Figure 1C. A coexpression heatmap indicated that the AKR1C3 expression level was tightly associated with the expression levels of MMR genes in GC (Fig. 1D).

### AKR1C3 is a potential indicator of GC prognosis

Because of its differential expression in the TCGA cohort, the diagnostic potential of AKR1C3 was further explored. A ROC curve was constructed based on the TCGA dataset; the AUC, sensitivity and specificity values were 0.593, 77.3% and 53.1%, respectively, when AKR1C3 was used as a potential indicator of GC (Fig. 1E). Kaplan‒Meier plotter showed that low AKR1C3 expression correlated with short overall survival time in GC patients in the TCGA cohort (*P*=0.049), as displayed in Figure 1F, which was also confirmed in the GSE14210 cohort (*P*=0.017, Fig. 1G). These results showed that tissue AKR1C3 is a potential indicator of GC prognosis.

### Construction and validation of the prognostic nomogram model

**Figure 2:**
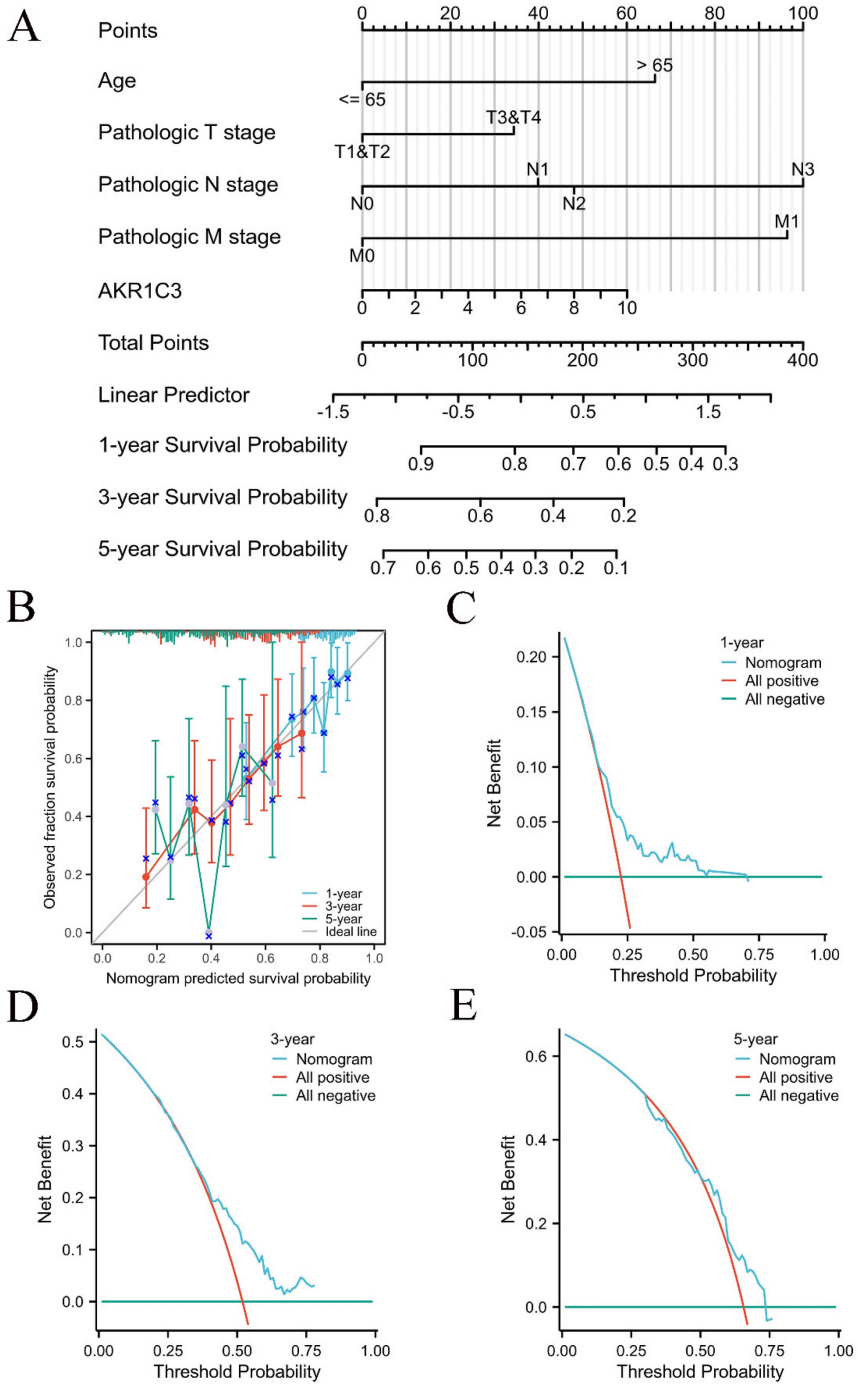
** Overall survival nomogram model and calibration plots.** A: Nomogram model for predicting the 1-, 3-, and 5-year OS of GC patients. B: Calibration plots of the overall survival nomogram model. C-E: The 1-, 3-, and 5-year DCA curves of the nomogram.

Considering the prognostic potential of tissue AKR1C3 expression in the TCGA cohort, univariable and multivariable Cox regression analyses were further performed to filter the prognostic risk factors in Supplementary Table 2. PH assumptions and VIF assumptions were made before nomogram construction, as shown in supplementary Table 3 and supplementary Table 4. Then, a prognostic nomogram model was established to predict the 1-, 3- and 5-year OS based on the results of multivariable Cox regression analysis (Fig. 2A). The concordance index (C-index) of the prognostic nomogram model was 0.644 (95% CI: 0.617-0.671). Calibration curves displayed a good calibration capability (Fig. 2B). In addition, the DCA curves suggested that our prognostic nomogram model was a practical model for clinical application (Fig. 2C-E).

### Validation of the clinical significance and prognostic value of AKR1C3 in GC

**Figure 3:**
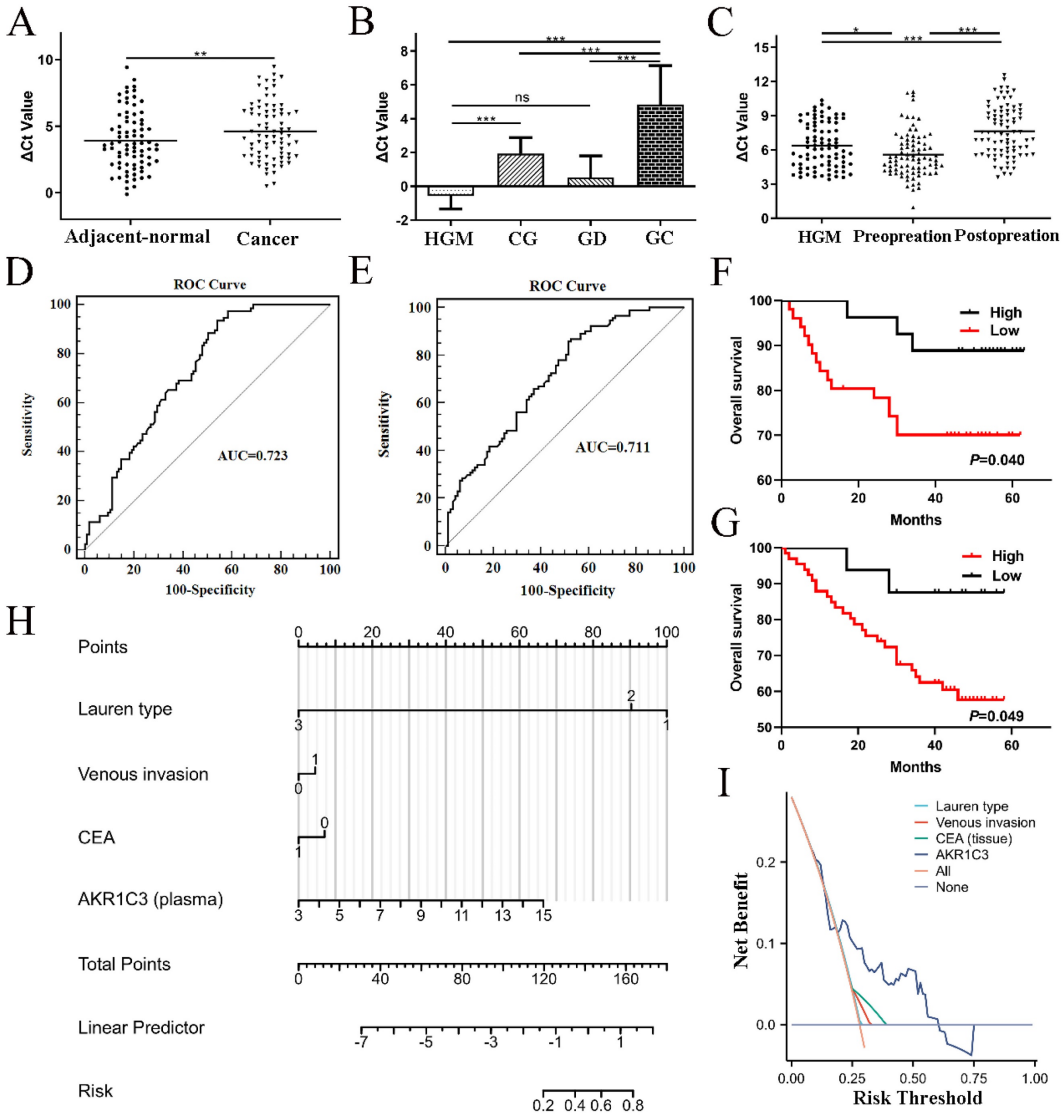
** Validation of the clinical significance and prognostic value of AKR1C3 in GC.** A: AKR1C3 expression was significantly downregulated in GC tissues. B: AKR1C3 levels in healthy gastric mucosa (HGM, *n*=37), chronic gastritis (CG, *n*=34), gastric dysplasia (GD, *n*=28) samples, and gastric cancer patients (*n*=78). AKR1C3 expression was prominently decreased in the GC group. C: AKR1C3 levels were significantly increased in the preoperation group (*n*=83) compared with the HGM group (*n*=83) but markedly decreased in the postoperation group (*n*=83) compared with the preoperation group. D: ROC curve of tissue AKR1C3 for distinguishing GC tissue samples from normal tissues. E: ROC curve of plasma AKR1C3 for distinguishing GC plasma samples from healthy plasma. F: Kaplan‒Meier survival plot showing that lower AKR1C3 expression in GC tissues was associated with poor survival time. G: Kaplan‒Meier survival plot showing that high plasma AKR1C3 levels correlated with poor prognosis in GC patients. H: Plasma nomogram construction for postoperative recurrence risk prediction. I: DCA curve of the nomogram model. (*P <0.05, **P<0.01, ***P<0.001).

AKR1C3 expression levels in 78 GC tissues and paired adjacent normal tissues were detected by PCR, which demonstrated that AKR1C3 expression was significantly downregulated in GC tissues (*P*=0.0034, Fig. 3A). Moreover, compared to the HGM, CG and GD groups, AKR1C3 expression in the GC group was also prominently decreased (*P*<0.001, Fig. 3B). In plasma, AKR1C3 levels significantly increased in the preoperation group compared with the HGM group (*P*=0.035) but markedly decreased in the postoperation group compared with the preoperation group (*P*<0.001, Fig. 3C). A ROC curve was constructed to investigate the potential value of AKR1C3 as an indicator of GC screening. As shown in Figure 3D, at the cutoff value, the AUC, sensitivity, and specificity values of the tissue ROC curve were 0.723, 0.936, and 0.461, respectively. Similarly, at the cutoff value, the AUC, sensitivity, and specificity values of the plasma ROC curve were 0.711, 0.868, and 0.474, respectively (Fig. 3E).

The Kaplan‒Meier survival plot showed that lower AKR1C3 expression in GC tissues was associated with poor survival time (Fig. 3F), whereas high plasma AKR1C3 levels correlated with a poor prognosis in GC patients (Fig. 3G), which was consistent with previous results.

Correlations between clinicopathological characteristics and AKR1C3 levels in GC tissues or plasma are listed in Table 1 and Supplementary Tables 5-6. Low expression of tissue AKR1C3 in GC was related to a larger diameter (*P*=0.005), advanced stage (*P*=0.042), higher invasion (*P*=0.034) and CA19-9 expression (*P*=0.029).

Analogously, a low plasma AKR1C3 level postoperation was correlated with the Lauren type (*P*=0.011), venous invasion (*P*=0.038) and CEA expression (*P*=0.034). Overall, the lower the level of AKR1C3 was in GC tissue, the worse the histopathological results, which is consistent with the patient's clinical prognosis results.

### Plasma nomogram construction for postoperative recurrence risk prediction

Based on the clinicopathological characteristics mentioned before, the AKR1C3 level in postoperative plasma was used as a biomarker to build a logistic nomogram to predict the risk of postoperative recurrence in GC patients (Fig. 3H). The C-index of the nomogram was 0.742 (95% CI: 0.611-0.872). DCA curves indicated that the model based on plasma AKR1C3 had an excellent prediction value (Fig. 3I).

### Annotation for AKR1C3 function in gastric carcinogenesis and metastasis from a multiomics perspective

**Figure 4:**
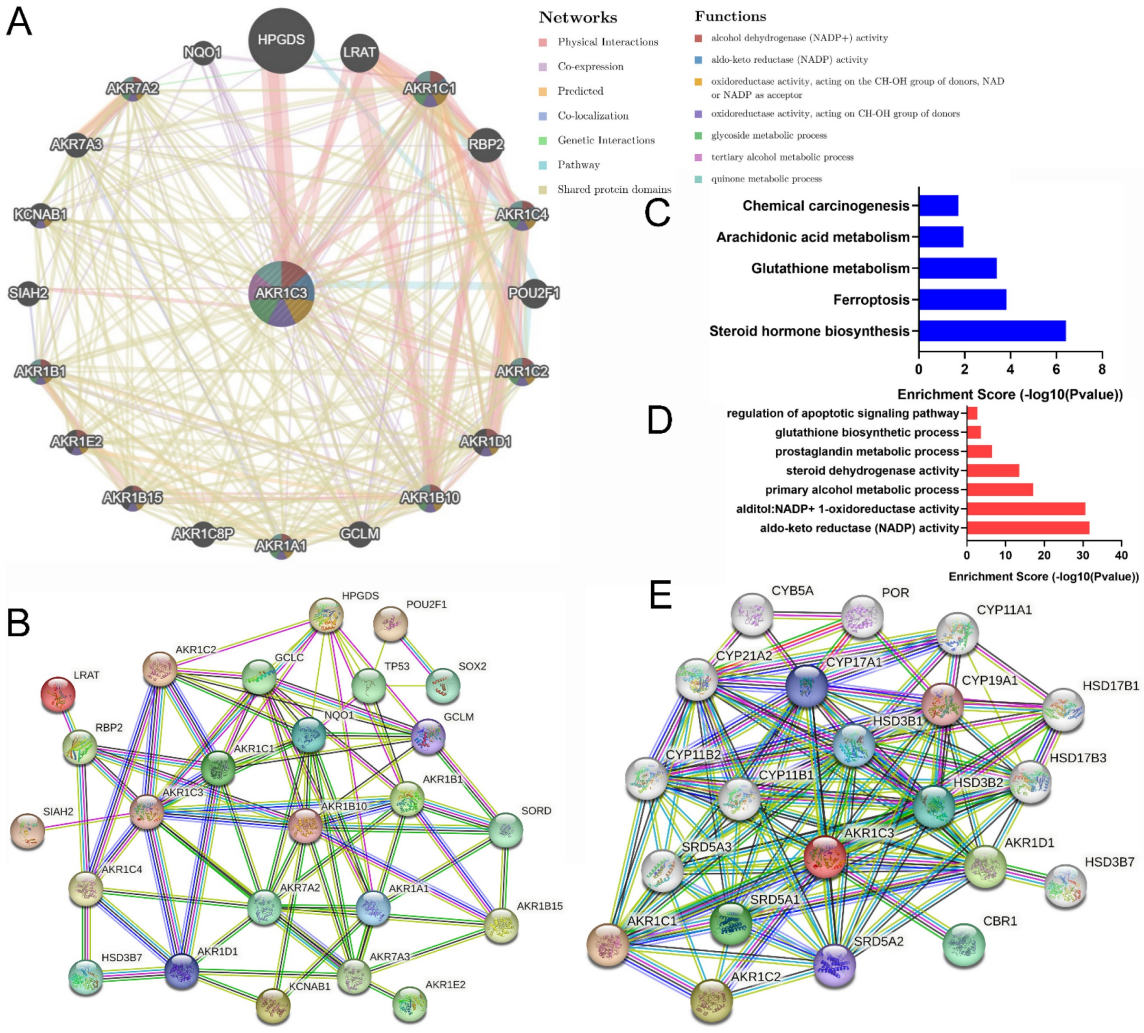
** Biological function analysis of genes and proteins interacting with AKR1C3.** A: Top 20 genes associated with *AKR1C3* (Centre) according to GeneMANIA. B: PPI network of 25 interacting proteins from the top 20 genes. C-D: KEGG (red) and GO (blue) analysis of 25 proteins in the PPI network. E: PPI network of interacting proteins of AKR1C3.

Gene interaction is a common mode for exerting biological regulatory functions. To analyze *AKR1C3* biological function at the pretranscriptional level, the interaction network was assessed at the gene level. GeneMANIA was used to explore the top 20 integrated gene pairs in Figure 4A. Then, proteins produced by these genes (AKR1A1, AKR1B1, AKR1B10, AKR1B15, AKR1C1, AKR1C2, AKR1C4, AKR1D1, AKR1E2, AKR7A2, AKR7A3, GCLC, GCLM, HPGDS, HSD3B7, KCNAB1, LRAT, NQO1, POU2F1, RBP2, SIAH2, SORD, SOX2, and TP53) were input into STRING to build the PPI network in Figure 4B. Finally, these protein molecules were subjected to KEGG analysis and GO classification. Our results showed that the expression of AKR1C3 was related to multiple biological functions and effects, such as NADP activity, ferroptosis, metabolic processes, steroid biosynthesis and carcinogenesis (Fig. 4C-D).

Protein interaction is an important way to exert functional effects. To probe the biological function of AKR1C3 during carcinogenesis and metastasis at the level of proteomics, AKR1C3 alone was input into STRING to establish the PPI network in Figure 4E, which included AKR1C1, AKR1C2, AKR1D1, CBR1, CYB5A, CYP11A1, CYP11B1, CYP11B2, CYP21A2, CYP17A1, CYP19A1, HSD17B1, HSD17B3, HSD3B1, HSD3B2, HSD3B7, POR, SRD5A1, SRD5A2, and SRD5A3. Similarly, the biofunctions of these factors were analyzed; they were mainly associated with metabolic processes, oxidative activity and steroid biosynthesis (Supplementary Figure 2).

**Figure 5:**
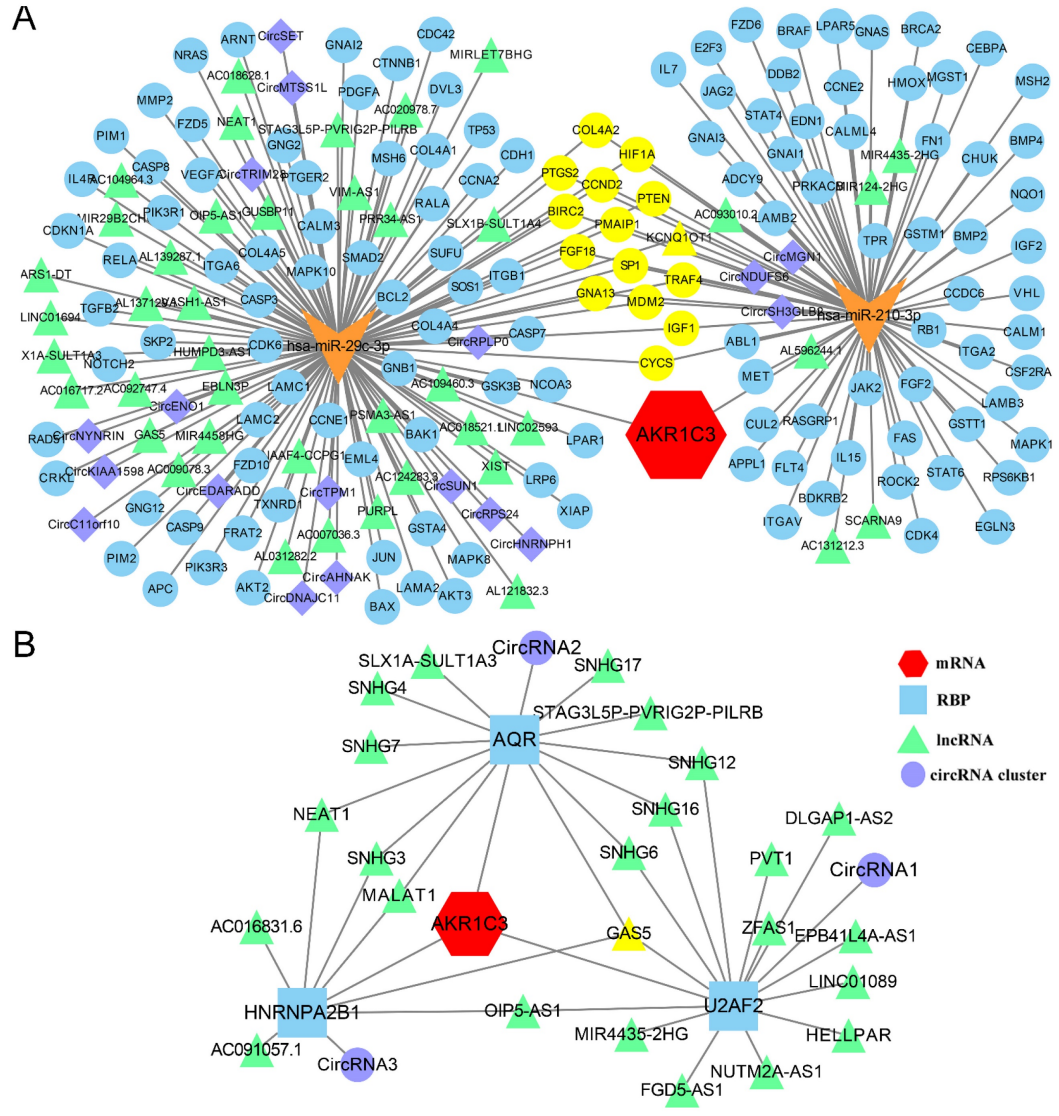
** CeRNA network and RBP-RNA regulatory network of AKR1C3.** A: CeRNA network of AKR1C3. Red represent AKR1C3 mRNA. Orange “V” represents miRNA. Green triangles represent lncRNAs. Purple rhombuses represent circRNAs. Blue circles represent mRNAs. Yellow represents co-regulated molecules. B: RBP-RNA regulatory network of AKR1C3. Yellow represents co-regulated molecules.

RNA interaction is an important regulatory mechanism, and the ceRNA mechanism is the basic mechanism by which these interactions affect cells. To probe the biological function of AKR1C3 during carcinogenesis and metastasis at the level of RNomics, a ceRNA network of AKR1C3 was constructed. The ceRNA network showed the interaction through miRNAs, mRNAs, lncRNAs and circRNAs. The interacting partners hsa-miR-29c-3p and hsa-miR-210-3p, validated in existing experiments, were acquired from TargetScan 8.0. Then, 138 mRNAs, 43 lncRNAs and 18 circRNAs were retrieved from Tarbase v8.0 and StarBase 3.0 according to the above criteria. The ceRNA network shown in Figure 5A revealed the potential downstream regulatory mechanism, which will provide a basis for further research to explore the regulatory mechanism of AKR1C3.

Interactions between RNA and protein can also have functional effects during carcinogenesis and metastasis. RBPs are vital links between ncRNAs and mRNAs; they can act as oncogenes or tumor suppressors in tumorigenesis. Given the central significance of RBPs, 3 stomach-related AKR1C3 mRNA-RBPs, 23 differentially expressed RBP-lncRNAs, and 3 circRNA clusters (4058 for circRNA1, 1594 for circRNA2, 8558 for circRNA3) were filtered from StarBase 3.0 according to the above methods. The RBP-RNA regulatory network displayed in Figure 5B demonstrates the detailed and complex relationships through different lenses, which will be helpful to clarify the intrinsic correlations between RNAs and proteins.

### Immune infiltration landscape analysis of AKR1C3

**Figure 6:**
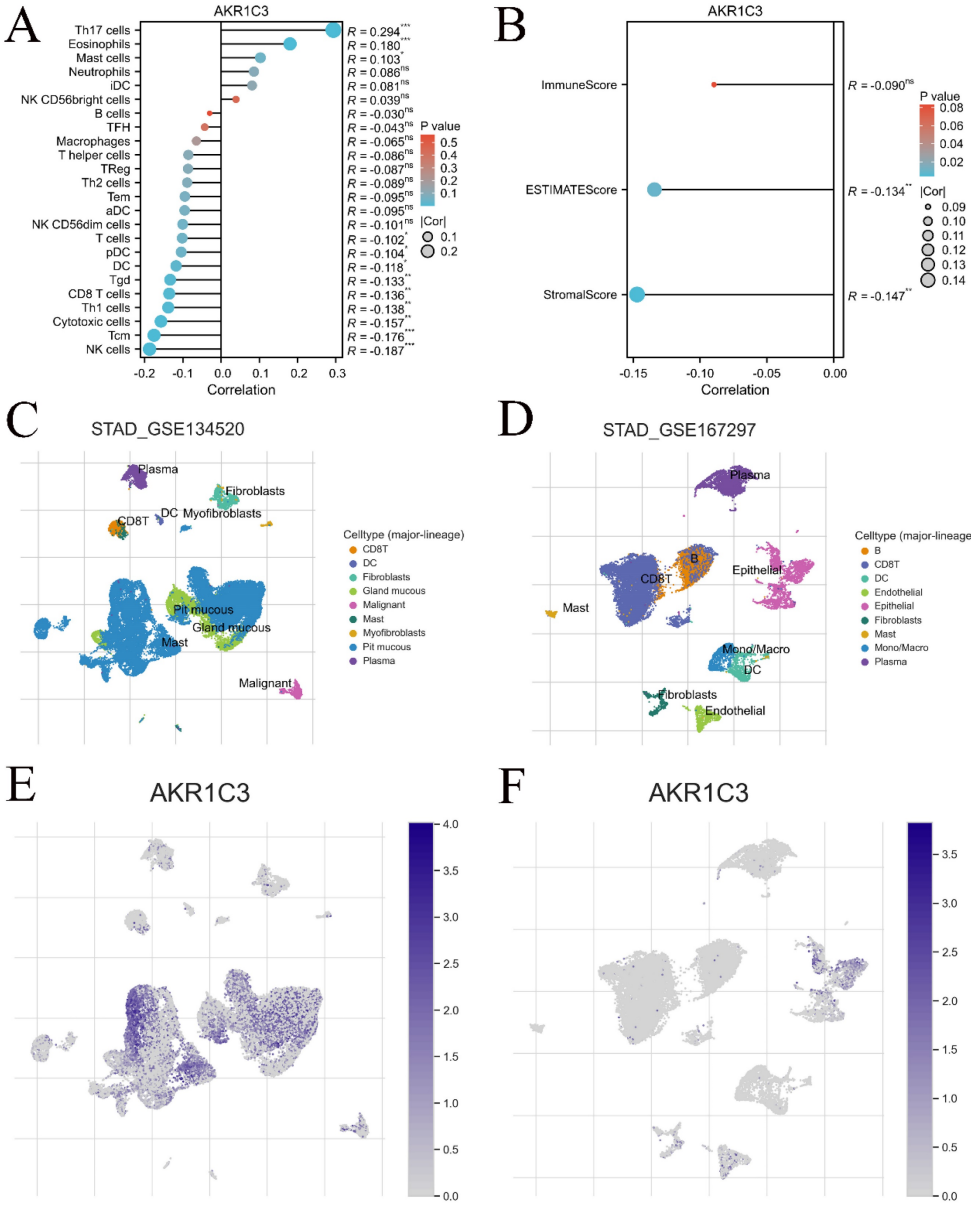
** Relationship between AKR1C3 expression level and immune cell infiltration.** A: Lollipop diagram of the relationship between AKR1C3 expression and immune cell infiltration levels in the TCGA cohort. B: The stromal score, immune score, and ESTIMATE score of different expression levels of AKR1C3 in GC samples. C: Annotation of major cell types in STAG_GSE134520. D: Annotation of major cell types in STAG_GSE167297. E: The proportion of patients with AKR1C3 expression in the STAG_GSE134520 cohort. F: The proportion of patients with AKR1C3 expression in the STAG_GSE167297 cohort (*P <0.05, **P <0.01, ***P<0.001).

To explore the impact of the immune cell landscape on the immunotherapy response, the infiltration levels of several immune cells were detected in the TCGA cohort. Our results showed that the expression of AKR1C3 was related to the infiltration level of many immune cells, such as Th17 cells, NK cells, eosinophils and mast cells (Fig. 6A), but negatively correlated with the stromal score and ESTIMATE score of GC samples in the TCGA cohort (Fig. 6B). Then, the relationship between AKR1C3 expression and the distribution of various immune cell types was compared using TISCH. Our results suggested that AKR1C3 in immune cells was mainly expressed in pit mucous in GSE134520 and epithelium in GSE167297 (Fig. 6C-F). Finally, associations between AKR1C3 expression and chemokines, immunoinhibitors, immunostimulators, and MHC molecules and drugs targeting AKR1C3 across cancers were displayed (Supplementary Figure 3), which revealed that AKR1C3 potentially interacts with 9 clinical drugs, including NADH (DB00157), indomethacin (DB00328), bimatoprost (DB00905), androstenedione (DB01536), rutin (DB01698), prostaglandin D2 (DB02056), flufenamic acid (DB02266), 2'-monophosphoadenosine 5'-diphosphoribose (DB03461), 3-carboxamido-1,3,5(10)-estratrien-17(R)-spiro-2'(5',5'-dimethyl-6'OXO) and tetrahydropyran (DB07700).

## Discussion

In the past several decades, GC has become one of the most prevalent cancers, imposing a heavy economic burden on the healthcare system and society 26. To date, screening for early GC and biotherapy for advanced GC in the clinic still face challenges. It is desirable to perform early detection and prompt treatment to promote remission and prevent relapse, further reducing the financial burden of GC 27.

AKR1C3 is a multifunctional molecule that is known as a prostaglandin F synthase and hormone activity regulator that regulates the occupancy of hormone receptors and cell proliferation in several cancers 28. For example, the abnormal expression of AKR1C3 affects the malignant potential of castration-resistant prostate cancer, and this effect can be inhibited by genistein 29. In hepatocellular carcinoma, AKR1C3 mediates the progression of ferroptosis by regulating the YAP/SLC7A11 signaling pathway, which is a novel treatment target for clinical therapy 30. Similarly, overexpression of AKR1C3 is associated with the development and aggressiveness of breast cancer, which induces resistance to anthracyclines and can be reversed by several specific inhibitors 31. However, its expression, biological functions and clinical significance in gastric carcinogenesis remain unclear.

Plasma is an important and convenient tool for addressing the dynamic changes in tumors in the clinic, which is an important part of “liquid biopsy” 32. Plasma biomarkers closely related to clinical and histopathological features are expected to become dynamic indicators reflecting cancer carcinogenesis, metastasis and recurrence. Hence, in our study, we detected and validated the AKR1C3 expression level in GC via TCGA cohort, GTEx cohort and clinical samples. Our results showed that AKR1C3 levels were decreased in GC tissue but significantly increased in GC plasma. The lower the level of AKR1C3 in GC tissue or the higher the level in plasma, the worse the histopathological results and patient's clinical prognosis, which implies that AKR1C3 is a promising biomarker for the screening and prognostic assessment of GC. Moreover, we have developed a novel nomogram for predicting the postoperative recurrence of GC based on patients' clinical characteristics and plasma AKR1C3 levels. This indicates the potential for the practical application of plasma AKR1C3 in clinical practice.

Emerging studies have confirmed that AKR1C3 can indicate chemotherapy responsiveness and immunotherapy in some cancers. Wu et al. noted that the AKR1C3-dependent increase in lipid droplet accumulation protects cancer cells from sorafenib-induced mitochondrial lipotoxicity and induces adaptation to sorafenib in hepatocellular carcinoma 33. AKR1C3 effectively catalyzes the activation of 11-oxygenated androgens in human peripheral blood mononuclear cells, which can decrease NK cell cytotoxicity and increase infection risk 34. Analogously, it has been revealed that single nucleotide polymorphisms of AKR1C3 alter drug metabolism and immune responses in aggressive non-Hodgkin lymphoma 35. However, the functional mechanism of dysregulated AKR1C3 during gastric carcinogenesis and metastasis is still poorly understood.

In this study, based on histopathological features and prognosis, we revealed the functional mechanism of AKR1C3 from a multiomics perspective. Multiomic functional analysis revealed that dysregulated AKR1C3 expression can widely participate in gene expression regulation through multiple regulatory modes at the gene, RNA and protein levels and exert various crucial biological effects on carcinogenesis and metastasis and related features and processes such as NADP activity, ferroptosis, and steroid biosynthesis. It has been revealed AKR1C1 and AKR1C3 can mediate cisplatin resistance in signet ring cell gastric carcinoma via autophagic cell death, which proves our point of view from the other side 36. Moreover, we found that AKR1C3 expression is correlated with the infiltration of several immune cell types and that AKR1C3 interacts with several clinical drugs. Our data provide a preclinical rationale for clinical trials of AKR1C3-targeted therapy.

Growing evidence indicates that ceRNA mechanism plays an indispensable role in regulating cell metabolism, function, and carcinogenesis in many types of tumors 37. Hence, we aimed to investigate the regulatory mechanism of AKR1C3 as a hub gene. We established AKR1C3-miRNA-mRNA/lncRNA/circRNA network and AKR1C3-RBP-lncRNA/circRNA network, which highlighted co-regulated molecules targeting several of the potential component-specific pathways. For example, Liu et al found that lncRNA GAS5 inhibits migration and invasion in GC via interacting with p53 protein, indicating GAS5 can become a potential therapeutic target in clinical application 38.

The limitations of the present study are that it includes an insufficient sample size for validation of the nomogram model and lacks functional verification results, which will be addressed in future studies.

## Conclusion

In conclusion, dysregulated AKR1C3 expression correlates with gastric carcinogenesis and immunotherapy response and is a promising biomarker and effective biotherapy target in GC.

## Supplementary Material

Supplementary figures and tables.

## Figures and Tables

**Table 1:** Correlation between clinicopathological characteristics and AKR1C3 levels in GC tissues.

Characteristics	Cases (%)	Δ*C*t Value	
		Mean±SD	*P* value
Age (year)			
> 65	52(66.67)	4.765±2.581	0.679
≤65	26(33.33)	4.529±1.882	
Sex			
Male	54(69.23)	4.608±2.537	0.663
Female	24(30.77)	4.863±1.945	
Tumor location			
Antrum	39(50.00)	5.125±2.485	0.425
Angle	11(14.10)	4.469±2.137	
Body	20(25.64)	4.136±2.114	
Others	8(10.26)	4.226±2.626	
Diameter (cm)			
≥5	36(46.15)	5.487±2.572	0.005
<5	42(53.85)	4.001±1.944	
Differentiation			
Well	14(17.95)	3.869±2.503	0.161
Moderate	34(43.59)	4.512±2.129	
Poor	30(38.46)	5.265±2.476	
TNM stage			
Early	20(25.64)	3.865±1.869	0.042
Advanced	58(74.36)	4.970±2.459	
Borrmann type			
I&II	5(8.62)	5.030±2.420	0.955
III&IV	53(91.38)	4.964±2.486	
Lauren type			
Intestinal	45(57.69)	4.345±2.156	0.137
Diffuse &Mixed	33(42.31)	5.152±2.576	
Invasion			
T_1_&T_2_	28(35.90)	3.868±1.974	0.034
T_3_&T_4_	50(64.10)	5.052±2.459	
lymphatic metastasis			
N_0_	30(38.46)	4.440±2.258	0.470
N_1-3_	48(61.54)	4.841±2.434	
Distal metastasis			
M_0_	69(88.46)	4.617±2.179	0.476
M_1_	9(11.54)	5.219±3.597	
Venous invasion			
Absent	41(52.56)	4.797±2.273	0.666
Present	37(47.44)	4.564±2.480	
Perineural invasion (PNI)			
Absent	33(42.31)	4.262±2.232	0.175
Present	45(57.69)	4.998±2.429	
CEA (tissue)			
Positive	70(89.74)	4.750±2.402	0.487
Negative	8(10.26)	4.133±2.020	
CA19-9 (tissue)			
Positive	45(57.69)	4.190±2.172	0.029
Negative	33(42.31)	5.363±2.471	

## References

[1] Sung H, Ferlay J, Siegel RL (2021). Global Cancer Statistics 2020: GLOBOCAN Estimates of Incidence and Mortality Worldwide for 36 Cancers in 185 Countries. CA Cancer J Clin.

[2] Arnold M, Park JY, Camargo MC (2020). Is gastric cancer becoming a rare disease?. A global assessment of predicted incidence trends to 2035. Gut.

[3] Yu X, Song X, Xie Y (2022). Establishment of an Absolute Quantitative Method to Detect a Plasma tRNA-Derived Fragment and Its Application in the Non-Invasive Diagnosis of Gastric Cancer. Int J Mol Sci.

[4] Tabari A, Chan SM, Omar OMF (2022). Role of Machine Learning in Precision Oncology: Applications in Gastrointestinal Cancers. Cancers (Basel).

[5] Xu Y, Zhang P, Zhang K (2021). The application of CA72-4 in the diagnosis, prognosis, and treatment of gastric cancer. Biochim Biophys Acta Rev Cancer.

[6] Smyth EC, Nilsson M, Grabsch HI (2020). Gastric cancer. Lancet.

[7] Wang F, Wei XL, Wang FH (2019). Safety, efficacy and tumor mutational burden as a biomarker of overall survival benefit in chemo-refractory gastric cancer treated with toripalimab, a PD-1 antibody in phase Ib/II clinical trial NCT02915432. Ann Oncol.

[8] Zhao Q, Cao L, Guan L (2019). Immunotherapy for gastric cancer: dilemmas and prospect. Brief Funct Genomics.

[9] Liu Y, Li C, Lu Y (2022). Tumor microenvironment-mediated immune tolerance in development and treatment of gastric cancer. Front Immunol.

[10] Li C, Zhao Y, Zheng X (2016). *In vitro* CAPE inhibitory activity towards human AKR1C3 and the molecular basis. Chem Biol Interact.

[11] Liu J, He P, Lin L (2019). Characterization of a highly specific monoclonal antibody against human aldo-keto reductase AKR1C3. Steroids.

[12] Penning TM, Byrns MC (2009). Steroid hormone transforming aldo-keto reductases and cancer. Ann N Y Acad Sci.

[13] Gao J, Aksoy BA, Dogrusoz U (2013). Integrative analysis of complex cancer genomics and clinical profiles using the cBioPortal. Sci Signal.

[14] Zhang L, Li X, Zhang J (2021). Prognostic Implication and Oncogenic Role of PNPO in Pan-Cancer. Front Cell Dev Biol.

[15] Germano G, Lamba S, Rospo G (2017). Inactivation of DNA repair triggers neoantigen generation and impairs tumour growth. Nature.

[16] A Marcell Szász, András L, Ádám N (2016). Cross-validation of survival associated biomarkers in gastric cancer using transcriptomic data of 1,065 patients. Oncotarget.

[17] Chi H, Jiang P, Xu K (2022). A novel anoikis-related gene signature predicts prognosis in patients with head and neck squamous cell carcinoma and reveals immune infiltration. Front Genet.

[18] Warde-Farley D, Donaldson SL, Comes O (2010). The GeneMANIA prediction server: biological network integration for gene prioritization and predicting gene function. Nucleic Acids Res.

[19] Szklarczyk D, Gable AL, Nastou KC (2021). The STRING database in 2021: customizable protein-protein networks, and functional characterization of user-uploaded gene/measurement sets. Nucleic Acids Res.

[20] McGeary SE, Lin KS, Shi CY (2019). The biochemical basis of microRNA targeting efficacy. Science.

[21] Tastsoglou S, Skoufos G, Miliotis M (2023). DIANA-miRPath v4.0: expanding target-based miRNA functional analysis in cell-type and tissue contexts. Nucleic Acids Res.

[22] Li J, Liu S, Zhou H (2014). starBase v2.0: decoding miRNA-ceRNA, miRNA-ncRNA and protein-RNA interaction networks from large-scale CLIP-Seq data. Nucleic Acids Res.

[23] Chi H, Xie X, Yan Y (2022). Natural killer cell-related prognosis signature characterizes immune landscape and predicts prognosis of HNSCC. Front Immunol.

[24] Sun D, Wang J, Han Y (2021). TISCH: a comprehensive web resource enabling interactive single-cell transcriptome visualization of tumor microenvironment. Nucleic Acids Res.

[25] Ru B, Wong C, Tong Y (2019). TISIDB: an integrated repository portal for tumor-immune system interactions. Bioinformatics.

[26] Shiratori Y, Hutfless S, Rateb G (2023). The burden of gastrointestinal diseases in Japan, 1990-2019, and projections for 2035. JGH Open.

[27] Casamayor M, Morlock R, Maeda H (2018). Targeted literature review of the global burden of gastric cancer. Ecancermedicalscience.

[28] Liu Y, He S, Chen Y (2020). Overview of AKR1C3: Inhibitor Achievements and Disease Insights. J Med Chem.

[29] Yu X, Yan J, Li Y (2023). Inhibition of castration-resistant prostate cancer growth by genistein through suppression of AKR1C3. Food Nutr Res.

[30] Chen J, Zhang J, Tian W (2023). AKR1C3 suppresses ferroptosis in hepatocellular carcinoma through regulation of YAP/SLC7A11 signaling pathway. Mol Carcinog.

[31] Liu Y, Chen Y, Jiang J (2023). Development of highly potent and specific AKR1C3 inhibitors to restore the chemosensitivity of drug-resistant breast cancer. Eur J Med Chem.

[32] Zhang P, Zhou X, He M (2019). Ultrasensitive detection of circulating exosomes with a 3D-nanopatterned microfluidic chip. Nat Biomed Eng.

[33] Wu C, Dai C, Li X (2022). AKR1C3-dependent lipid droplet formation confers hepatocellular carcinoma cell adaptability to targeted therapy. Theranostics.

[34] Schiffer L, Bossey A, Kempegowda P (2021). Peripheral blood mononuclear cells preferentially activate 11-oxygenated androgens. Eur J Endocrinol.

[35] Gustafson HL, Yao S, Goldman BH (2014). Genetic polymorphisms in oxidative stress-related genes are associated with outcomes following treatment for aggressive B-cell non-Hodgkin lymphoma. Am J Hematol.

[36] Phoo NLL, Dejkriengkraikul P, Khaw-On P (2021). Transcriptomic Profiling Reveals AKR1C1 and AKR1C3 Mediate Cisplatin Resistance in Signet Ring Cell Gastric Carcinoma via Autophagic Cell Death. Int J Mol Sci.

[37] Wu S, Zhong B, Yang Y (2023). ceRNA networks in gynecological cancers progression and resistance. J Drug Target.

[38] Liu Y, Yin L, Chen C (2020). Long non-coding RNA GAS5 inhibits migration and invasion in gastric cancer via interacting with p53 protein. Dig Liver Dis.

