# Supplementary material for: Defining the biological functions and clinical significance of AKR1C3 in gastric carcinogenesis through multiomics functional analysis and immune infiltration analysis
Source: J Cancer. 2024 Mar 17;15(9):2646–58. doi: 10.7150/jca.94228 (PMC10988316; doi:10.7150/jca.94228)
Supplement: Supplementary file 1 — Supplementary figures and tables. [file jcav15p2646s1.zip › Supplementary Table.docx]

**Supplementary Table. 1 Correlation between AKR1C3 expression levels in gastric cancer tissues and clinicopathological parameters in the TCGA cohort**

| **Characteristics** | **Low expression of AKR1C3** | **High expression of AKR1C3** | ***P* value** |
| --- | --- | --- | --- |
| N (%) | 187 | 188 |  |
| Age |  |  | 0.318 |
| **≤** 65 | 77 (20.8%) | 87 (23.5%) |  |
| > 65 | 108 (29.1%) | 99 (26.7%) |  |
| Sex |  |  | 0.639 |
| Male | 118 (31.5%) | 123 (32.8%) |  |
| Female | 69 (18.4%) | 65 (17.3%) |  |
| Histological type |  |  | 0.621 |
| Diffuse type | 34 (9.1%) | 29 (7.8%) |  |
| Mucinous type | 11 (2.9%) | 8 (2.1%) |  |
| Not otherwise, specified | 102 (27.3%) | 105 (28.1%) |  |
| Papillary type | 4 (1.1%) | 1 (0.3%) |  |
| Signet Ring type | 5 (1.3%) | 6 (1.6%) |  |
| Tubular type | 31 (8.3%) | 38 (10.2%) |  |
| Pathologic T stage |  |  | 0.471 |
| T_1_ | 11 (3%) | 8 (2.2%) |  |
| T_2_ | 34 (9.3%) | 46 (12.5%) |  |
| T_3_ | 87 (23.7%) | 81 (22.1%) |  |
| T_4_ | 51 (13.9%) | 49 (13.4%) |  |
| Pathologic N stage |  |  | 0.256 |
| N_0_ | 60 (16.8%) | 51 (14.3%) |  |
| N_1_&N_2_&N_3_ | 117 (32.8%) | 129 (36.1%) |  |
| Pathologic M stage |  |  | 0.335 |
| M_0_ | 165 (46.5%) | 165 (46.5%) |  |
| M_1_ | 10 (2.8%) | 15 (4.2%) |  |
| Histologic grade |  |  | **0.016** |
| G1 | 4 (1.1%) | 6 (1.6%) |  |
| G2 | 56 (15.3%) | 81 (22.1%) |  |
| G3 | 123 (33.6%) | 96 (26.2%) |  |
| *H. pylori* infection |  |  | 0.598 |
| Yes | 8 (4.9%) | 10 (6.1%) |  |
| No | 74 (45.4%) | 71 (43.6%) |  |
| Pathologic stage |  |  | 0.164 |
| Stage I | 27 (7.7%) | 26 (7.4%) |  |
| Stage II | 55 (15.6%) | 56 (15.9%) |  |
| Stage III | 82 (23.3%) | 68 (19.3%) |  |
| Stage IV | 13 (3.7%) | 25 (7.1%) |  |

**Supplementary Table. 2 Univariable and multivariable Cox regression analysis of risk factors**

| **Characteristics** | **Total**  **(N)** | **Univariate analysis** | |  | **Multivariate analysis** | |
| --- | --- | --- | --- | --- | --- | --- |
|  |  | **Hazard ratio (95% CI)** | ***P*** |  | **Hazard ratio (95% CI)** | ***P*** |
| Pathologic T stage | 362 |  | **0.008** |  |  |  |
| T_1_&T_2_ | 96 | Reference |  |  | Reference |  |
| T_3_&T_4_ | 266 | 1.719 (1.131-2.612) | **0.011** |  | 1.315 (0.827-2.091) | 0.248 |
| Pathologic N stage | 352 |  | **<0.001** |  |  |  |
| N_0_ | 107 | Reference |  |  | Reference |  |
| N_1_ | 97 | 1.629 (1.001-2.649) | **0.049** |  | 1.444 (0.854-2.441) | 0.170 |
| N_2_ | 74 | 1.655 (0.979-2.797) | **0.060** |  | 1.558 (0.902-2.691) | **0.111** |
| N_3_ | 74 | 2.709 (1.669-4.396) | **<0.001** |  | 2.499 (1.479-4.222) | **<0.001** |
| Age | 367 |  | **0.005** |  |  |  |
| <= 65 | 163 | Reference |  |  | Reference |  |
| > 65 | 204 | 1.620 (1.154-2.276) | **0.005** |  | 1.824 (1.268-2.622) | **0.001** |
| Sex | 370 |  | 0.182 |  |  |  |
| Female | 133 | Reference |  |  |  |  |
| Male | 237 | 1.267 (0.891-1.804) | 0.188 |  |  |  |
| Pathologic M stage | 352 |  | **0.010** |  |  |  |
| M_0_ | 327 | Reference |  |  | Reference |  |
| M_1_ | 25 | 2.254 (1.295-3.924) | **0.004** |  | 2.301 (1.277-4.146) | **0.006** |
| Histologic grade | 361 |  | 0.169 |  |  |  |
| G1 | 10 | Reference |  |  |  |  |
| G2 | 134 | 1.648 (0.400-6.787) | 0.489 |  |  |  |
| G3 | 217 | 2.174 (0.535-8.832) | 0.278 |  |  |  |

Entry 0.1; Removal: 0.15

**Supplementary Table 3 PH assumptions of the prognostic nomogram.**

| **Characteristics** | **χ^2^** | ***P*** |
| --- | --- | --- |
| Age | 0.00099017 | 0.9749 |
| Pathologic T stage | 0.065459 | 0.7981 |
| Pathologic N stage | 2.3014 | 0.5122 |
| Pathologic M stage | 4.7526 | 0.0293 |
| AKR1C3 | 0.16922 | 0.6808 |
| GLOBAL | 7.2812 | 0.4002 |

**Supplementary Table. 4 VIF assumptions of the prognostic nomogram.**

| **Characteristics** | **VIF** |
| --- | --- |
| Age |  |
| <= 65 | Reference |
| > 65 | 1.0662 |
| Pathologic T stage |  |
| T_1_&T_2_ | Reference |
| T_3_&T_4_ | 1.13 |
| Pathologic N stage |  |
| N_0_ | Reference |
| N_1_ | 1.9228 |
| N_2_ | 1.7602 |
| N_3_ | 1.9896 |
| Pathologic M stage |  |
| M_0_ | Reference |
| M_1_ | 1.1146 |
| AKR1C3 | 1.0445 |

**Supplementary Table. 5 Correlation between AKR1C3 levels and clinicopathological parameters in preoperative plasma samples.**

| **Characteristics** | **Cases (%)** | **Δ*C*t Value** |  |
| --- | --- | --- | --- |
|  |  | **Mean±SD** | ***P* value** |
| Age (y) |  |  |  |
| > 65 | 51(68.00) | 5.635±2.030 | 0.518 |
| ≤ 65 | 24(32.00) | 5.310±1.999 |  |
| Sex |  |  |  |
| Male | 52(69.33) | 5.574±2.030 | 0.781 |
| Female | 23(30.67) | 5.433±2.013 |  |
| Tumor location |  |  |  |
| Antrum | 40(53.33) | 5.761±2.263 | 0.213 |
| Angle | 15(20.00) | 5.255±1.516 |  |
| Body | 2(2.67) | 7.765±0.629 |  |
| Others | 18(24.00) | 5.003±1.697 |  |
| Diameter (cm) |  |  |  |
| ≥ 5 | 41(54.67) | 5.860±1.648 | 0.121 |
| < 5 | 34(45.33) | 5.135±1.717 |  |
| Differentiation |  |  |  |
| Well | 4(5.33) | 5.050±1.213 | 0.516 |
| Moderate | 33(44.00) | 5.289±1.998 |  |
| Poor | 38(50.67) | 5.792±2.092 |  |
| TNM stage |  |  |  |
| Early | 17(22.67) | 5.202±2.010 | 0.447 |
| Advanced | 58(77.33) | 5.628±2.020 |  |
| Borrmann type |  |  |  |
| I&II | 16(27.59) | 5.926±1.854 | 0.536 |
| III&IV | 42(72.41) | 5.551±2.101 |  |
| Lauren type |  |  |  |
| Intestinal | 35(46.67) | 5.236±1.946 | 0.237 |
| Diffuse&Mixed | 40(53.33) | 5.789±2.057 |  |
| Invasion |  |  |  |
| T_1_&T_2_ | 25(33.33) | 5.217±1.773 | 0.342 |
| T_3_&T_4_ | 50(66.67) | 5.688±2.121 |  |
| Lymphatic metastasis |  |  |  |
| N_0_ | 28(37.33) | 5.472±2.030 | 0.845 |
| N_1-3_ | 47(62.67) | 5.566±2.023 |  |
| Distal metastasis |  |  |  |
| M_0_ | 61(81.33) | 5.607±2.040 | 0.500 |
| M_1_ | 14(18.67) | 5.201±1.923 |  |
| Venous invasion |  |  |  |
| Absent | 41(54.67) | 5.380±1.857 | 0.478 |
| Present | 34(45.33) | 5.714±2.199 |  |
| Perineural invasion (PNI) |  |  |  |
| Absent | 37(49.33) | 5.671±2.316 | 0.556 |
| Present | 38(50.67) | 5.395±1.686 |  |
| CEA (tissue) |  |  |  |
| Positive | 57(76.00) | 5.421±1.762 | 0.402 |
| Negative | 18(24.00) | 5.880±2.688 |  |
| CA19-9 (tissue) |  |  |  |
| Positive | 31(41.33) | 5.782±1.930 | 0.841 |
| Negative | 44(58.67) | 5.571±2.089 |  |

**Supplementary Table. 6 Correlation between AKR1C3 levels in postoperative plasma samples and clinicopathological parameters.**

| **Characteristics** | **Cases (%)** | **Δ*C*t Value** |  |
| --- | --- | --- | --- |
|  |  | **Mean±SD** | ***P* value** |
| Age (y) |  |  |  |
| > 65 | 51(68.00) | 7.806±2.264 | 0.584 |
| ≤ 65 | 24(32.00) | 7.485±2.545 |  |
| Sex |  |  |  |
| Male | 52(69.33) | 7.672±2.371 | 0.864 |
| Female | 23(30.67) | 7.774±2.288 |  |
| Tumor location |  |  |  |
| Antrum | 40(53.33) | 7.863±2.365 | 0.503 |
| Angle | 15(20.00) | 7.807±2.809 |  |
| Body | 2(2.67) | 9.300±2.447 |  |
| Others | 18(24.00) | 7.085±1.864 |  |
| Diameter (cm) |  |  |  |
| ≥ 5 | 41(54.67) | 7.962±2.459 | 0.297 |
| < 5 | 34(45.33) | 7.391±2.194 |  |
| Differentiation |  |  |  |
| Well | 4(5.33) | 6.980±2.533 | 0.186 |
| Moderate | 33(44.00) | 7.229±1.971 |  |
| Poor | 38(50.67) | 8.191±2.572 |  |
| TNM stage |  |  |  |
| Early | 17(22.67) | 6.839±2.226 | 0.084 |
| Advanced | 58(77.33) | 7.957±2.336 |  |
| Borrmann type |  |  |  |
| I&II | 16(27.59) | 8.673±2.281 | 0.151 |
| III&IV | 42(72.41) | 7.768±2.326 |  |
| Lauren type |  |  |  |
| Intestinal | 35(46.67) | 6.973±2.064 | **0.011** |
| Diffuse & Mixed | 40(53.33) | 8.343±2.412 |  |
| Invasion |  |  |  |
| T_1_&T_2_ | 25(33.33) | 7.391±2.241 | 0.419 |
| T_3_&T_4_ | 50(66.67) | 7.859±2.402 |  |
| lymphatic metastasis |  |  |  |
| N_0_ | 28(37.33) | 7.024±2.297 | 0.052 |
| N_1-3_ | 47(62.67) | 8.108±2.302 |  |
| Distal metastasis |  |  |  |
| M_0_ | 61(81.33) | 7.613±2.330 | 0.487 |
| M_1_ | 14(18.67) | 8.099±2.457 |  |
| Venous invasion |  |  |  |
| Absent | 41(54.67) | 7.193±2.228 | **0.038** |
| Present | 34(45.33) | 8.318±2.311 |  |
| Perineural invasion (PNI) |  |  |  |
| Absent | 37(49.33) | 7.882±2.464 | 0.519 |
| Present | 38(50.67) | 7.530±2.241 |  |
| CEA (tissue) |  |  |  |
| Positive | 57(76.00) | 7.382±2.333 | **0.034** |
| Negative | 18(24.00) | 8.722±2.135 |  |
| CA19-9 (tissue) |  |  |  |
| Positive | 31(41.33) | 7.554±1.799 | 0.647 |
| Negative | 44(58.67) | 7.808±2.679 |  |
