# Supplementary material for: Defining the biological functions and clinical significance of AKR1C3 in gastric carcinogenesis through multiomics functional analysis and immune infiltration analysis
Source: J Cancer. 2024 Mar 17;15(9):2646–58. doi: 10.7150/jca.94228 (PMC10988316; doi:10.7150/jca.94228)
Supplement: Supplementary file 1 — Supplementary figures and tables. [file jcav15p2646s1.zip › Supplementary Figure.docx]

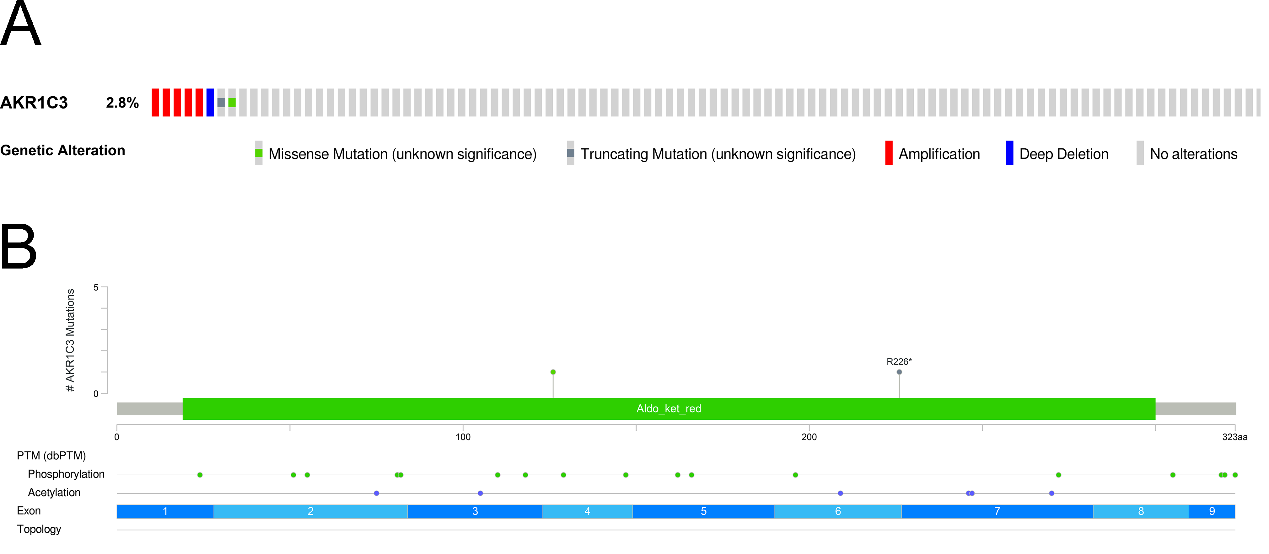


Supplementary Figure 1 The mutation landscape of the *AKR1C3* gene.


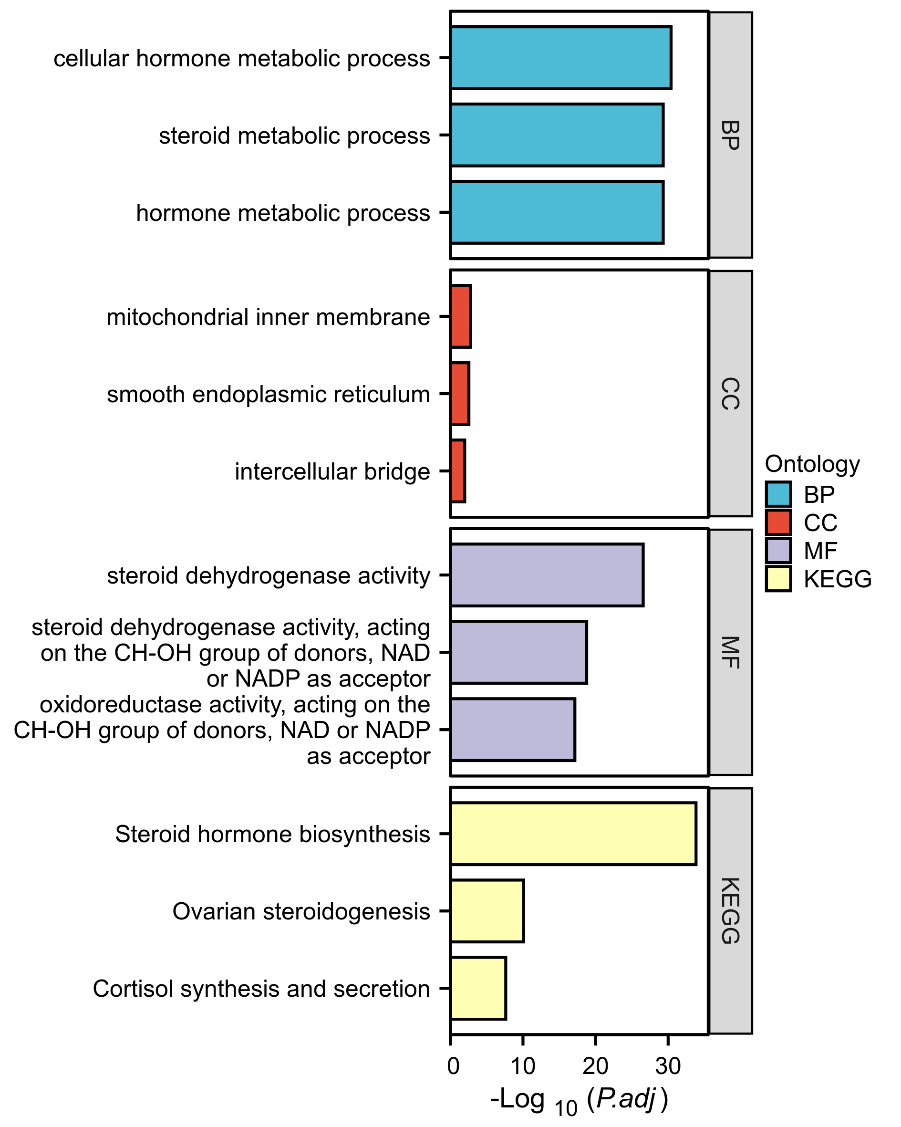
A: Summary of *AKR1C3* mutation alterations. B: The mutation types, numbers, and sites of *AKR1C3* genetic alterations.

Supplementary Figure 2 KEGG and GO analysis.

KEGG and GO analysis of the AKR1C3-related PPI network (BP: biological process; MF: molecular function; CC: cellular component).


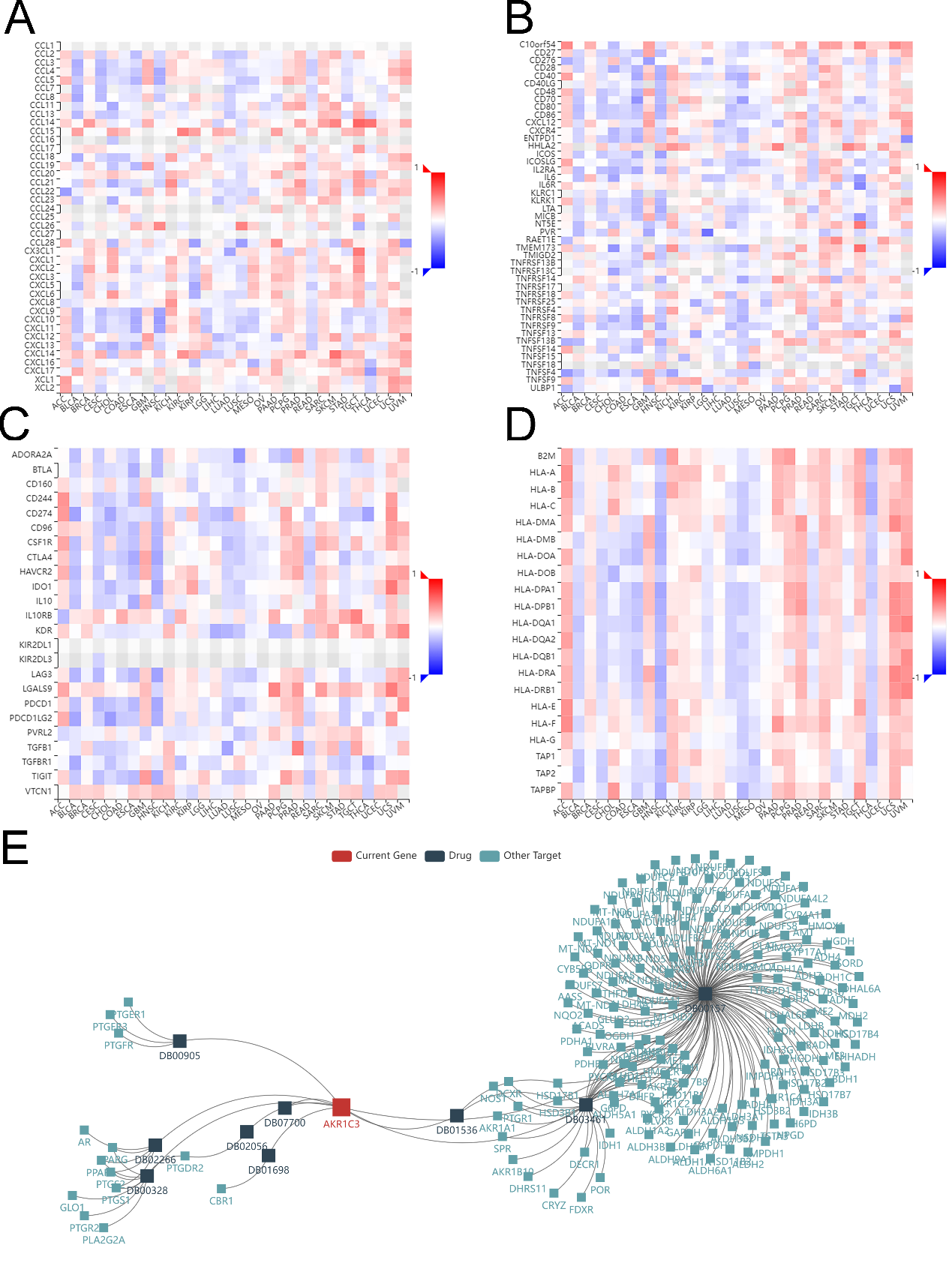


Supplementary Figure 3 The immune landscape of AKR1C3 across cancers.

The associations between AKR1C3 expression and chemokines (A), immunoinhibitors (B), immunostimulators (C), and MHC molecules (D) across cancers. E: The association between AKR1C3 and target drugs.
